# The protective effect of Buzhong Yiqi decoction on ischemic stroke mice and the mechanism of gut microbiota

**DOI:** 10.3389/fnins.2022.956620

**Published:** 2022-12-15

**Authors:** Qianqian Li, Mengxin Cao, Zijun Wei, Jianing Mei, Yuechan Zhang, Man Li, Manlin Li, Yunyun Zhang, Zhifei Wang

**Affiliations:** ^1^Department of Internal Neurology, Yueyang Hospital of Integrated Traditional Chinese and Western Medicine Affiliated to Shanghai University of Traditional Chinese Medicine, Shanghai, China; ^2^School of Basic Medicine, Shanghai University of Traditional Chinese Medicine, Shanghai, China; ^3^Institute of Traditional Chinese Medicine, Shanghai University of Traditional Chinese Medicine, Shanghai, China

**Keywords:** Buzhong Yiqi decoction (BZYQD), MCAO, ischemic stroke, gut microbiota, mouse

## Abstract

Buzhong Yiqi decoction (BZYQD) has been developed for preventing or reducing the recurrence of ischemic stroke for a long time in China. However, the mechanism of action of the BZYQD is not completely understood. Our research aims to determine whether the mechanism of action of BZYQD is by regulating gut microbiota using 16SR RNA and fecal microbiota transplantation. In a cerebral ischemia mouse model, the results showed that prophylactic administration of BZYQD could reduce brain infarct volume and improve neurological function and behavior. The prophylactic administration of BZYQD could regulate intestinal microbiota and increase the abundance of butyrate-producing Prevotellaceae_NK3B31_group and probiotic *Akkermansia* in mice 72 h after surgery. Transplanting BZYQD-administered bacterial flora into antibiotic-depleted mice could reproduce the therapeutic effects of BZYQD. Overall, our study provided molecular insights into the mechanism and impact of BZYQD in the prevention of cerebral ischemic damage and highlighted the potential of regulation of intestinal microbiota as a therapeutic approach for ischemic stroke.

## Introduction

Ischemic stroke is the necrosis of ischemic and hypoxic cerebral tissue caused by various reasons. It has attracted worldwide attention because of its high morbidity rate, high mortality rate, high recurrence rate, and extensive sequelae (GBD 2016 Stroke Collaborators, 2019). One of the characteristics of ischemic stroke is the necrosis and apoptosis of a large number of cells around ischemia, resulting in different degrees of cell death and damage (Feng et al., 2017), in which necrosis begins within hours, and apoptosis occurs within hours to days. Apoptotic cells that occur in the ischemic penumbra have impaired function but cell metabolism still occurs, and taking measures to reduce apoptotic cells can effectively avoid tissue continuation infarction and damage to adjacent tissues. Therefore, anti-apoptosis is an important method for therapeutic intervention (Ji et al., 2014; Zaro-Weber et al., 2019; Tang et al., 2020). Thrombolysis and thrombectomy, which are the main treatment methods for ischemic stroke, can only be applied to a small number of people due to the limited time window (Yoshimura et al., 2022). The utilization of neuroprotective drugs has mostly failed in clinical trials. Traditional Chinese medicine has obvious advantages in comprehensive diagnosis and treatment and overall regulation, especially in anti-apoptosis (Cheng et al., 2012; Gao et al., 2015; Li et al., 2017), which provides space for the development of traditional Chinese medicine.

The gut microbiota is closely related to human health and disease. Intestinal microbes can achieve two-way communication through nerves, endocrine, immunity, metabolism, and brain, which is called the “microbiota–gut–brain axis” (Cryan et al., 2020). For ischemic stroke, it promotes intestinal dysfunction and disturbs the gut microbiota (Singh et al., 2016; Stanley et al., 2016; Kurita et al., 2020), and an impaired intestinal microbiota can lead to increased translocation of commensal bacteria, infection (Stanley et al., 2016), and inflammation. Metabolite production induces neuroinflammatory responses (Guo et al., 2021) and stimulates immune responses in brain tissue through intestinal immune cells (Benakis et al., 2016), thereby worsening stroke outcomes. This emphasizes the importance of the homeostasis of gut microbiota, suggesting that the correction of disturbed gut ecology may be an important direction for the treatment of stroke. Recent studies have shown that traditional Chinese medicine treatments, such as Tongqiao Huoxue Decoction, Tanhuo decoction, and Nao Mai Tong can interfere with ischemic stroke in the result of intestinal flora improvement (Zhang et al., 2020; Guo et al., 2021; Lin et al., 2021). BZYQT comes from the “Spleen and Stomach Treatise” written by Zhang Zhongjing, an ancient Chinese medical sage. BZYQD can replenish Qi and raise Yang, regulate Qi and purify spleen, and treat internal injuries caused by deficiency and labor. In our clinical study, BZYQD can be used to prevent the recurrence of patients with post-stroke and relieve stroke sequelae (Yu et al., 2018), whose pharmacological mechanism involves anti-inflammatory and bacteriostatic, anti-apoptotic, repairing damaged intestinal mucosa, and regulating intestinal flora (Xing-zhong et al., 2008; Yushi et al., 2019; Jingwen, 2020). Studies have shown that BZYQD fermented preparations shape the intestinal flora of broilers and promote the production performance of broilers by increasing *Lactobacillus* and decreasing *Escherichia coli* (Shuang-xing et al., 2019). BZYQD can also ameliorate the growth of intestinal probiotics in mice with diarrhea to improve diarrhea and repair the disordered state of intestinal bacteria (Wei, 2013). The role of BZYQD needs to be further explored, whether it has a neuroprotective influence on stroke mice, and whether it can play a role by regulating the intestinal microbiota of mice. Accordingly, we employed the transient middle cerebral artery infarction model (MCAO) to study the consequence of preventive administration of BZYQD on ischemic stroke and its effect on gut microbiota.

## Materials and methods

### Animals

All animal procedures were conducted in accordance with guidelines for using experimental animals and approved by the Animal Research Committee of the Laboratory Animal Center of Shanghai University of Traditional Chinese Medicine (PZSHUTCM18110603). Seven-week-old C57BL/6J male mice mainly weighing 22–25 g were obtained from Shanghai Weitong Lihua and were maintained in a standard specific pathogen-free (SPF) barrier environment, including a 12/12 light–dark cycle, a temperature setting of 22°C ± 3°C, and a 55–70% humidity environment. Litter was changed weekly and mice had free access to food and water. Female mice were not used in this research to avoid the effects of sex hormones. All mice that died postoperatively were excluded from the data.

### Preparation of Buzhong Yiqi decoction samples

The prescription came from Li Dongyuan’s “Spleen and Stomach Theory,” which included 15 g of Astragalus, 12 g of Atractylodes, 6 g of tangerine peel, 3 g of Cimicifuga, 3 g of Bupleurum, 12 g of Codonopsis, 6 g of Zhigancao, and 9 g of Angelica. The herbs in BZYQD (Table 1) were identified by the Department of Pharmacy of Yueyang Hospital affiliated with the Shanghai University of Traditional Chinese Medicine in accordance with the standard scheme. In conformity with the standard method of the Chinese Pharmacopoeia, all raw herbs of BZYQT were immersed in 10 volumes of distilled water for 0.5 h, decocted for 1 h using a low flame after boiling, with eight layer gauze filter liquid. The filter residue of the medicinal herbs was decocted in eight volumes of water for 1 h and then the solution was filtered again (Wang et al., 2020). The individual herbs were prepared using the same extraction method. All crude drugs were stored at room temperature and BZYQT concentrated decoction solution was stored at –20°C.

**TABLE 1:** Mobile phase gradient elution procedure.

Time/Min.	A/%	B/%	Time/Min.	A/%	B/%
0	4	96	65	30	70
5	4	96	70	30	70
10	7.5	92.5	95	50	50
25	15	85	100	50	50
30	15	85	105	95	5
45	20	80	110	95	5
50	20	80			

Buzhong Yiqi decoction extracting solution (1 ml) was accurately added to a 2 ml volumetric flask and dissolved to the tick mark with methanol. After centrifugation, the supernatant was filtered through a 0.45 μm membrane filter before high purity liquid chromatography (HPLC) analysis. The extraction solution of each single flavor medicine in the same method was obtained as a control medicinal material for testing. Medicinal material standards including mullein isoflavones, saikosaponin d, isovaleric acid, ferulic acid, glycyrrhizic acid, hesperidin, were purchased from the Institute of Chinese Materia Medica of Shanghai University of Traditional Chinese Medicine and dissolved by weight into their respective standard solutions for the chemical identification of the main peaks of the fingerprint.

### High purity liquid chromatography analysis of Buzhong Yiqi decoction

The Agilent 1100 HPLC system and diode array instrument were used to analyze various chromatographic systems. The chromatographic analysis was carried out at 30°C using a Welch Ultimate XB-C18 column (4.6 mm × 250 mm, 5 μm) and aqueous acetonitrile (B) acetonitrile (A) –0.05% phosphate (B) as the mobile phase to elute at a flow rate gradient of 1.0 ml/min with detecting wavelengths of 254 and 280 nm[26]. The gradient elution procedure settings are shown in Table 1.

### Experimental grouping and intervention methods

#### Trial I

To determine the optimal dosage, different doses of BZYQT were administered by MCAO 3 days after the operation. The dose–dose experiment was conducted by setting up the sham operation group (sham), operation control group (MCAO), high-dose group (B-high, 8 g/kg), and low-dose group (B-low, 4 g/kg). The results showed that the high-dose group had a smaller infarct size (Supplementary Figure 1) and a better improvement trend in neurological function and behavior than the low-dose group (Supplementary Figure 1). Therefore, the high-dose group was selected for follow-up experiments.

#### Trial II

To observe the neuroprotective effect and intestinal microbiota mechanism of BZYQD in mice with ischemic stroke, the experiment was divided into two groups: the BZYQD group and the control group (MCAO). The BZYQD group was given an intragastric administration of 1 ml/10 g of BZYQD two times a day for 14 days, and the MCAO group was given water by gavage. After 14 days of preventive administration, MCAO was performed, and the effect of BZYQD on ischemia-reperfusion was observed at 72 h after the acute phase.

#### Trial III

The fecal bacteria transplantation method was used to explore whether the intestinal flora of BZYQD-administered mice was the key point. The experiment was divided into three groups: BZYQD microflora transplantation group (BZYQT-FMT), the control microflora transplantation group (MCAO-FMT), and the transplantation control group (MCAO). We mixed three antibiotics to deplete the mouse microflora, including metronidazole 50 mg, vancomycin 25 mg, and ciprofloxacin 10 mg in a ratio of 5:2.5:1 (Freitag et al., 2019; Li et al., 2021) into 100 ml of mouse drinking water for free drinking.

The process of microbiota transplantation is as follows (Singh et al., 2016). Mice were prophylactically administered BZYQT/sterile water for 14 days as transplant donors (BZYQT flora and control group). During the bacterial colony transplantation process, 1 g of mouse fecal pellets after 14 days were collected, homogenized with 1 ml of sterile water, and centrifuged at 2,000 rpm for 10 min. We gavage the supernatant into the recipient mice after centrifugation, 200 μl/mice, and the bacterial colony transplantation was completed within 1 h.

### Middle cerebral artery infarction model

Transient middle cerebral artery focal cerebral ischemia was induced by using the intraluminal filament model of MCAO, as described elsewhere (Li et al., 2020). The Reward Small Animal Inhalation Anesthesia Machine was used to induce 2% isoflurane and 1.5% anesthesia maintenance during the operation. Intraoperative mice rectal temperature was dynamically monitored maintaining at 37 ± 0.5°C with a heating pad. The mice’s right common carotid artery was exposed through an incision in the midline of the neck, and a 0.20–0.21 mm silicone monofilament was passed through the common carotid artery into the internal carotid artery to obstruct the middle cerebral artery for 45 min. After the infarction, the blood flow in the brain region of the mice was detected by a Doppler flowmeter, which was maintained at about 30% of the preoperative level. After 45 min of infarction, the filament was removed to allow for reperfusion, and the common carotid artery was ligated to close the wound. Postoperatively, the mice were placed in an incubator at 35–37°C until the mice were awake. Sham operation group mice underwent the same procedure, in addition to inserting and removing the filament immediately.

### Cerebral infarct volume

Under anesthesia, mice were sacrificed 72 h after MCAO, and 2,3,5-triphenyltetrachloride (TTC) (Sigma-Aldrich) staining was performed on harvested brains to assess infarct volume. After freezing the brain at –20°C, we cut it into six 1 mm slices on the coronal surface, and each slice is incubated with 2% TTC at 37°C for 10 min. Normal brain tissue was stained red, while cerebral infarction tissue was pale because the dehydrogenase activity decreased due to ischemia-hypoxic necrosis and could not react with TTC. In the image analysis software, ImageJ was used to measure the infarct volume which was calculated by integrating the infarct area of all brain slices.

### Neurological sore

The neurological deficits were assessed by the modified neurological severity score (mNSS) at 24, 48, and 72 h after reperfusion (Dou et al., 2021). This score assesses nerve damage including motor, sensory, and neurological reflexes. Specifically, it includes front paw flexion, hind paw flexion, head turning in a fixed direction, inability to walk in a straight line, paralyzed lateral rotation, unilateral hemiplegia, grasping ability, vision, corneal reflex, pinna reflex, startle reflex, and exploration ability. Each test is scored as 0 points for normality and 1 point for abnormality, with a total score of 12 points. The higher the score, the greater the degree of neurological impairment.

### Rotarod

After the stroke, a rotarod was used to assess motor balance and coordination after the stroke. Mice were accelerated from 5 to 40 rpm on a rotating rod set at a constant speed of 4 min, recording the time it takes for each mouse to fall from the spin rod and comparing the average time of the day test. Mice were acclimated for 3 days prior to MCAO surgery, running five times on day 1 and then three times per day thereafter, ensuring that the mice reached a baseline of approximately 200 s on the bar. Mice were formally trained three times a day for 24, 48, and 72 h after surgery, with an average time of three times recorded.

### Terminal-deoxynucleoitidyl transferase mediated nick end labeling

Apoptosis was detected previously using a terminal-deoxynucleoitidyl transferase mediated nick end labeling (TUNEL) staining kit (Roche) (Zhang et al., 2019). Mice were perfused with cardiac bloodstream for 5 min using fresh 4°C PBS and 4% paraformaldehyde. After dehydration, the brain tissue was sliced in a coronal plane with a thickness of 25 mm per piece using a frozen slicer. Brain slices from the optic chiasm were fixed using 4% paraformaldehyde, digested with protease K for 10 min at room temperature, and incubated with a TUNEL mixture for 1 h at 37°C. The sections were counterstained with DAPI for 5 min and placed under Nikon A1-Si confocal microscopy (Nikon, Tokyo, Japan) to observe brain apoptotic cells under 20× microscopy. Five random areas of cortex and striatum near the ischemic penumbra near the optic chiasma were found under the microscope to calculate the percentage of apoptotic cells/nuclei.

### 16S rRNA gene sequencing analysis

All materials used for the fecal collection were sterilized in an autoclave. Fecal samples were collected from each mouse into 2 ml Eppendorf tubes, labeled, and stored in a –80°C freezer for cryopreservation until 16S rRNA gene sequencing analysis. The collected feces were purified by DNA extraction and PCR amplification and sequenced on the Illumina platform. The collected feces were purified by DNA extraction and PCR amplification and sequenced on the Illumina platform. The quality of the obtained sequence was modified by the UPARSE platform (Edgar, 2013) to obtain the final OTU sequence, which was compared with the SILVA database to obtain the bacteria corresponding to the OTU. On the MiSeq platform (^1^mothur v. 1.47.0) (Kozich et al., 2013), the sobs index, Shannon index, and Simpson index can be obtained according to the different calculation instructions, which are used to represent the Alpha diversity of species sex. The species evolution tree was constructed according to the maximum likelihood method on the FastTree platform (version 2.1.3, ^2^), and the R language (version 3.3.1) was used to draw the graph. The LEfSe multi-level species difference discriminant analysis was performed on the GitHub platform, and the samples were subjected to linear discriminant analysis (LDA) according to the taxonomic composition according to different grouping conditions, to find out the communities or species that had a significant difference in the sample division.

### Statistical analysis

Statistical analysis of data was performed using GraphPad Prism 8.0.2 and the R language software package. Data were described as mean ± SD. Two-group data analysis was used for Student’s *t*-test, multi-group analysis was used for one-way ANOVA, and multi-group multi-time point analysis was used for two-way ANOVA. Statistical comparisons of intestinal flora were assessed using the Kruskal–Wallis H test and the Mann–Whitney U test in R software. The *p*-value of <0.05 was considered statistically significant.

## Results

### High-performance liquid analysis of Buzhong Yiqi decoction

**FIGURE 1:**
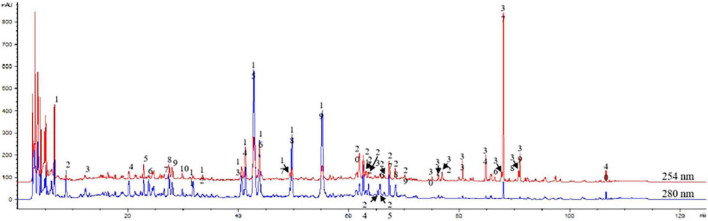
Assessment of Buzhong Yiqi decoction (BZYQD) quality using a multi-component high purity liquid chromatography (HPLC) fingerprint. The picture shows the typical chromatogram of BZYQD at 254 and 280 nm.

The fusion fingerprint is shown in Figure 1. According to the retention time and UV spectrum, and referring to the chromatographic characteristics of the reference solution, peak 13 was ferulic acid, peak 14 was isoferulic acid, peak 19 was hesperidin, peak 29 was calycosin, peak 36 was glycyrrhizic acid, and peak 38 was saposaponin d. Chromatographic profiles of BZYQD and control medicinal materials were analyzed and compared (Table 2). For 20–73 min, the main components in this area included organic acids and flavonoids. Peaks 9, 11, 17, 18, and 19 were the characteristic peak groups of Tangerine peel; peaks 20, 28, 29, and 35 were the characteristic peak groups of Astragalus; peaks 7, 12, and 13 were the characteristic peak groups of Angelica sinensis. Peaks 23 and 38 were the characteristic peaks of Bupleurum, and peaks 8, 16, 21, 26, and 27 were the characteristic peaks of cohosh. After 74–106 min of fusion fingerprint, this region was mainly saponins with strong UV absorption, and peak 36 was glycyrrhizin (licorice saponin). In addition, we analyzed the common peaks corresponding to the fingerprints and identified them (Table 2). BZYQD fingerprint determination method is reliable and reproducible, which meets the requirements of fingerprint determination.

**TABLE 2:** The table shows the attribution of the common peaks in the fingerprint.

Peak number	1	2	3	4	5	6	7	8	9	10
Time of appearance	6.650	8.730	12.299	20.131	22.772	23.700	26.484	27.378	27.963	29.818
Medicinal material attribution	Astragalus, Atractylodes, Codonopsis, Angelica, tangerine peel, Bupleurum	Atractylodes, Codonopsis, tangerine peel	Atractylodes, Codonopsis, tangerine peel	Astragalus, Atractylodes, Codonopsis, Angelica, tangerine peel, Bupleurum	Tangerine peel	Licorice	Angelica, Licorice	Cimicifuga	Tangerine peel	Tangerine peel, Licorice

**Peak number**	**11**	**12**	**13**	**14**	**15**	**16**	**17**	**18**	**19**	**20**

Time of appearance	31.733	33.480	40.507	41.182	42.714	43.749	49.241	49.630	55.115	61.842
Medicinal material attribution	Tangerine peel	Astragalus, Atractylodes Codonopsis, Angelica, Bupleurum	Angelica, tangerine peel, Licorice, Cimicifuga	Astragalus	Licorice	Cimicifuga	Tangerine peel	Tangerine peel	Tangerine peel	Astragalus

**Peak number**	**21**	**22**	**23**	**24**	**25**	**26**	**27**	**28**	**29**	**30**

Time of appearance	62.562	63.005	63.520	65.115	65.579	66.317	67.226	68.296	70.079	74.977
Medicinal material attribution	Cimicifuga	Licorice	Bupleurum	Licorice	Licorice	Cimicifuga	Cimicifuga	Astragalus	Astragalus	Licorice

**Peak number**	**31**	**32**	**33**	**34**	**35**	**36**	**37**	**38**	**39**	**40**

Time of appearance	76.144	76.798	80.513	84.692	85.639	87.582	87.904	90.635	90.895	106.457
Medicinal material attribution	Tangerine peel	Licorice	Licorice	Licorice	Astragalus	Licorice	Licorice	Bupleurum	Licorice	Licorice

### Neuroprotective effect of prophylactic Buzhong Yiqi decoction administration against cerebral ischemia injury

**FIGURE 2:**
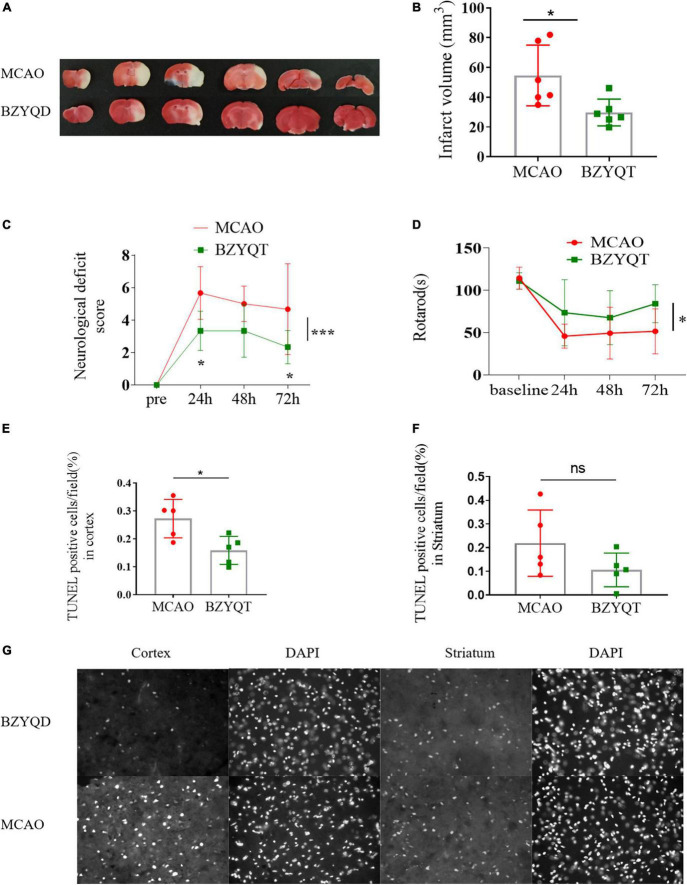
Inhibition of focal ischemic strokes caused by middle cerebral artery obstruction Through BZYQD Preventive Therapy. **(A)** Representative images and **(B)** quantitation of infarct volume in TTC-stained coronal sections of mouse brains with a 45-min MCAO followed by 72 h of reperfusion. **(C)** Modified neurological severity score (mNSS) test was evaluated before and 24, 48 and 72 h after MCAO using the modified neurological score (mNSS). **(D)** Analysis of motion and balance function by rotarod test before and 24, 48 and 72 h after MCAO. The numerical assessment of terminal-deoxynucleoitidyl transferase mediated nick end labeling (TUNEL)-positive cells in the cortex **(E)** and striatum **(F)** near the cerebral infarction. **(G)** The representative photographs of apoptosis-positive cells using TUNEL staining in the cerebral cortex (cortex) and striatum (striatum) of the BZYQT group and the con group, and DAPI is the nucleus of the same part of the field of view. All statistical graphs: *p < 0.05, ***p < 0.0005. Data are presented as x^–^ ± s, *n* = 6.

After 14 days of preventive BZYQD administration, we performed MCAO molding and then administered it for 3 days after perfusion to observe the protective effect of tonic Yiqi soup on mice in strokes. Quantification of infarct volume with 2,3,5-triphenyltetrazolium blue (TTC) staining showed that prophylactic treatment with BZYQD could significantly reduce the volume of infarctions in mice (Figures 2A,B, *P* = 0.0212). Treatment with BZYQD reduced mNSS score at 24 and 72 h after MCAO and significantly improved neurological impairment compared with the control group (Figure 2C, *p* = 0.0328, *p* = 0.0328). We identified motor dysfunction conditions in mice by rotarod and BZYQD treatment had an impact on the time spent on the rotarod (Figure 2D, *P* = 0.0470). During cerebral ischemia, the main cell damage in the ischemic penumbra is apoptosis, and the rescue of penumbra cells is the key link of treatment. We performed TUNEL staining in different experimental groups to detect the degree of apoptosis of cells near the cerebral infarction area. BZYQD treatment had no significant effect on apoptotic cells in the striatum (Figure 2E), but it attenuated apoptosis in the cortical region near cerebral infarction in mice induced by MCAO reperfusion (Figure 2F, *P* = 0.0175). The results showed that BZYQD treatment could rescue apoptotic cells in the ischemic penumbra cells in the cerebral infarction area and alleviate the effects of MCAO surgery on mice.

### Influence of the prophylactic administration of Buzhong Yiqi decoction on the intestinal flora of mice

To assess whether treatment and behavioral differences were associated with changes in gut microbiota diversity and bacterial relative abundance in fecal samples, we sequenced the V3–V4 region of the 16S rRNA gene from intestinal microbiota collected before prophylactic administration (0 day), 14 days after prophylactic administration (14 days), and 72 h after reperfusion (72 h).

**FIGURE 3:**
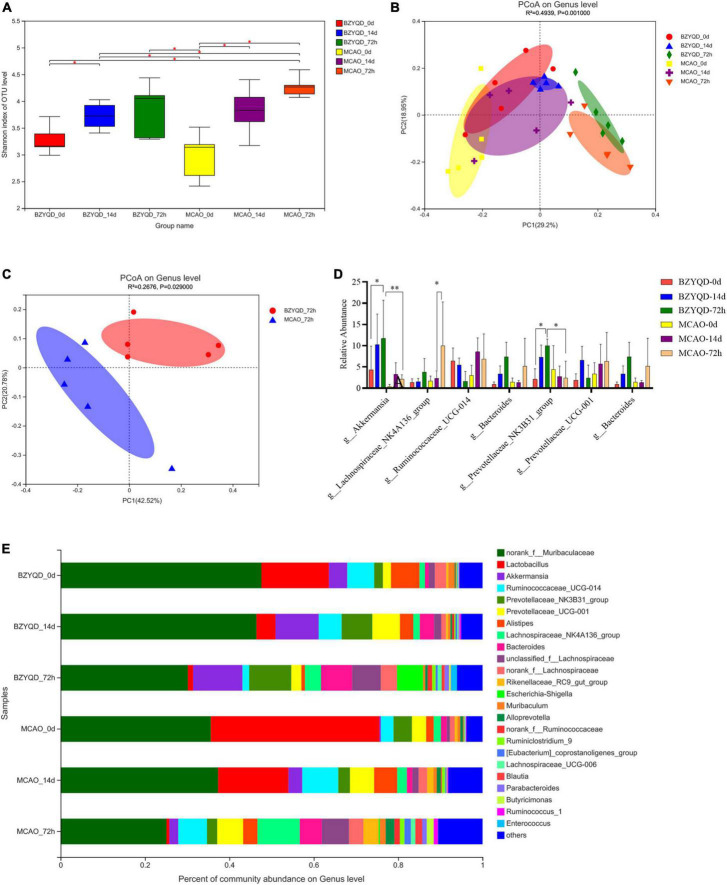
The effect of the prophylactic administration of BZYQT on the intestinal flora of mice. **(A)** Sobs index analysis represents Alpha diversity. **(B)** Analysis of species composition of intestinal flora in two groups at the genus level at three time points. **(C)** PCoA analysis of intestinal flora 72 h after operation of the two groups. **(D)** The species composition of the gut microbiota at the genus level at three time points in the two groups was analyzed. **(E)** Relative abundance analysis of major genera at the genus level. *P < 0.05. Data are expressed as mean ± standard deviation. *N* = 5.

According to the alpha diversity index estimation, there was no statistically significant difference in intestinal microbial diversity between the two groups (Figure 3A). We used PCoA analysis based on the Bray–Curtis distance matrix algorithm to identify potential principal components that influenced the differences in the community composition of the samples (Figure 3B). After 72 h of reperfusion, the two groups of mice were located far away from the other time points. As shown in Figure 3, the total variation observed at the PC1 level was 42.52% (Figure 3C, *P* = 0.029), and the total variation observed at the PC2 level was 20.78%, indicating that there was a certain difference between the two groups of bacterial. Therefore, we obtained that the BZYQD group was significantly different from the MCAO group in 72 h postoperative microbial community structure.

There was no statistical significance in the analysis of community composition between the two groups on day 0 (untreated healthy mice), and the two groups were considered to be the same at baseline (Figures 3D,E). After 14 days of prophylactic treatment, the abundance of Firmicutes in the BZYQD group was less than that in the MCAO group at the phylum level (Figure 3E, 18.71 ± 4.81 vs 38.16 ± 6.05%, *P* = 0.038). At the genus level, eight bacteria were increased and one bacterium was decreased in the BZYQD group and the MCAO group compared with their 0 days genera. At 72 h after MCAO, at the phylum level, Firmicutes increased and Bacteroidetes decreased in both groups. Compared with the 14 days phylum in the MCAO group, the BZYQD group had more changes in the phylum, with the increase of *Proteobacteria* and the decrease of *Bacteroidetes, Patescibac-te-ria*, unclassified_k__norank_d__Bacteria, *Tenericutes*, and *Cyanobacteria*, but there was no statistical significance. At the genus level, seven bacteria in the BZYQD group decreased and nine bacteria increased. In the MCAO group, 13 bacteria increased and two bacteria decreased. The bacterial community after 72 h of MCAO was compared with that after 14 days of prophylactic administration. The norank_f__ Roche and *Lactobacillus* were decreased in both groups. Unclassified_f__ Lachnospira, norank_f__ Lachnospira, and Brautia, *Lachnospiraceae*_UCG-006, and *Escherichia coli*–*Shigella* all increased. We believe that these changes may be the manifestation of intestinal dysbiosis after a stroke. Notably, after reperfusion, *Akkermansia* (Figure 3D, 11.701 ± 8.989% vs 4.336 ± 5.573%, *P* = 0.404) and Prevotella_NK3B31_group (9.99 ± 1.573 vs 2.086 ± 2.515%, *P* = 0.0216) were significantly higher than that of 0 day, and there was a statistical difference compared with the MCAO group (Figure 3D, *P* = 0.0023, *p* = 0.0320). Compared with the control group, the administration of BZYQD increased SCFA-producing bacteria in mice after MCAO reperfusion, which may be the key point of beneficial effect.

### The improvement effect of intestinal flora on stroke in mice administered with Buzhong Yiqi decoction

We explored whether the beneficial outcomes of BZYQD prophylactic administration were mediated by gut microbiota by means of microbiota transplantation. Before transplantation, we used metronidazole, vancomycin, and ciprofloxacin mixed antibiotic drinking water to deplete the intestinal flora of mice, mainly depleting broad-spectrum gram-negative bacteria, gram-positive bacteria, and anaerobic bacteria. After MCAO modeling, the feces of mice after prophylactic administration were transplanted into mice after reperfusion, and the effect of short-term flora transplantation on mice with acute phase transient cerebral ischemia was observed.

**FIGURE 4:**
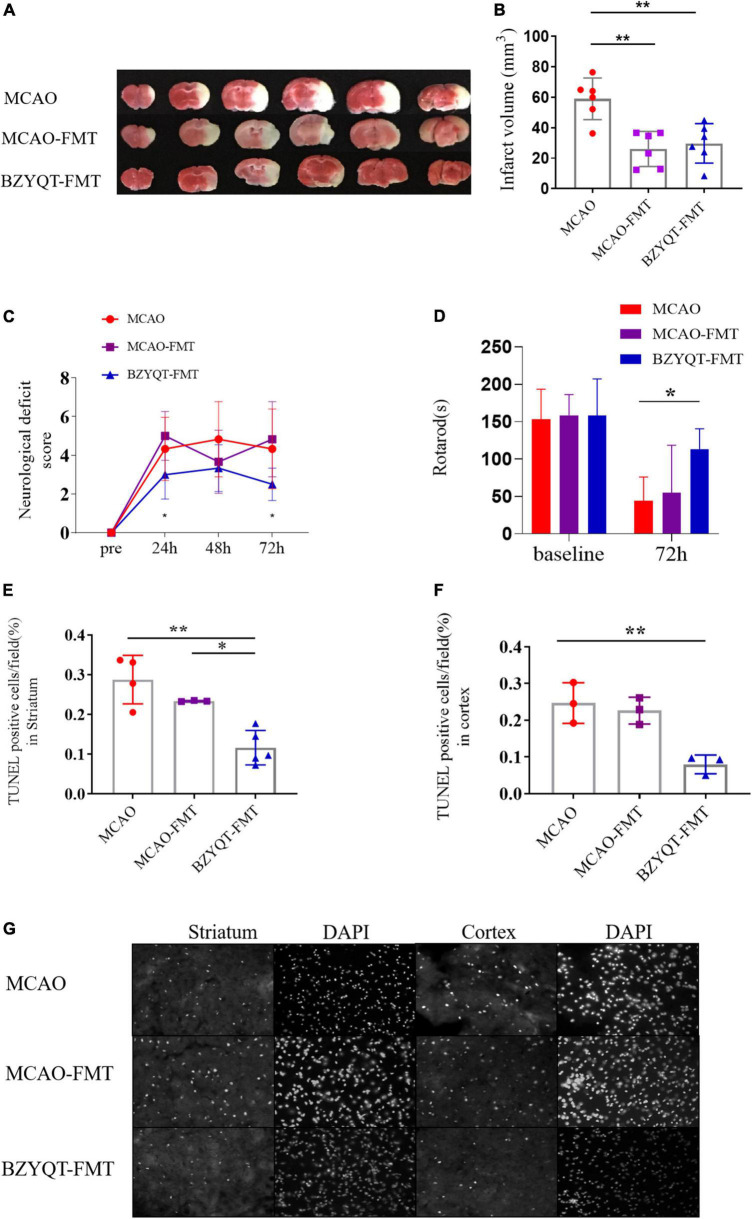
Improvement of ischemic stroke by microbiota transplantation. Panel **(A)** is the volume of cerebral infarction at 72 h after the operation (*n* = 6). Panel **(B)** is the ImageJ quantitative analysis of cerebral infarction volume. **(C)** Compare analysis of neurological functional deficits before and 24, 48, and 72 h after MCAO. (*n* = 6). Panel **(D)** is the rotarod motor function of mice 72 h after operation (*n* = 6). Panel **(E)** is the statistics of apoptotic cells in the cortex near the ischemic penumbra detected by the TUNEL kit (*n* = 3). Panel **(F)** is the statistics of apoptotic cells in the striatum near the cerebral infarction (*n* = 4/3/5). Panel **(G)** is the analysis of apoptosis-positive cells in the cortex and striatum near the cerebral infarction. Five images of different fields of view were collected at each site, and the ratio of apoptosis-positive cells/nuclei was counted. *p < 0.05 and **p < 0.01. Data are expressed as mean ± standard deviation.

The results showed that both BZYQD-FMT and MCAO-FMT could reduce the volume of cerebral infarction compared with the MCAO group (Figures 4A,B, *p* = 0.0498, *p* = 0.0196). There was no significant difference between the BZYQD-FMT group and the MCAO-FMT group. The neurological function scores of the BZYQD-FMT group were significantly better than those of the MCAO-FMT group at 24 and 72 h (Figure 4C, *P* = 0.0049, *P* = 0.0369). In addition, the exercise time of the mice in the BZYQD-FMT group on the rotarod was significantly increased, which was statistically significant compared with the MCAO group (Figure 4D, *P* = 0.0205), and showed a significant improvement trend compared with the MCAO-FMT group (Figure 4D, *P* = 0.0566). These results suggest that the BZYQD-FMT group has neuroprotective effects.

By comparing the apoptotic cells near the ischemic penumbra of mice in different groups, including the cortex and striatum, it was found that the apoptotic cells in the cortex and striatum of the BZYQD-FMT group were significantly less than those of the MCAO group (Figures 4E–G, *P* = 0.0048, *P* = 0.0009), in which the apoptotic cells in the vicinity of the striatum in the BZYQD-FMT group were significantly higher than those in the MCAO-FMT group (Figure 4E, *P* = 0.0160). This indicates that the BZYQD-FMT group can inhibit the apoptosis of penumbra cells, which is related to the improvement of neurological function.

### The effect of microbiota transplantation on the intestinal flora of mice

**FIGURE 5:**
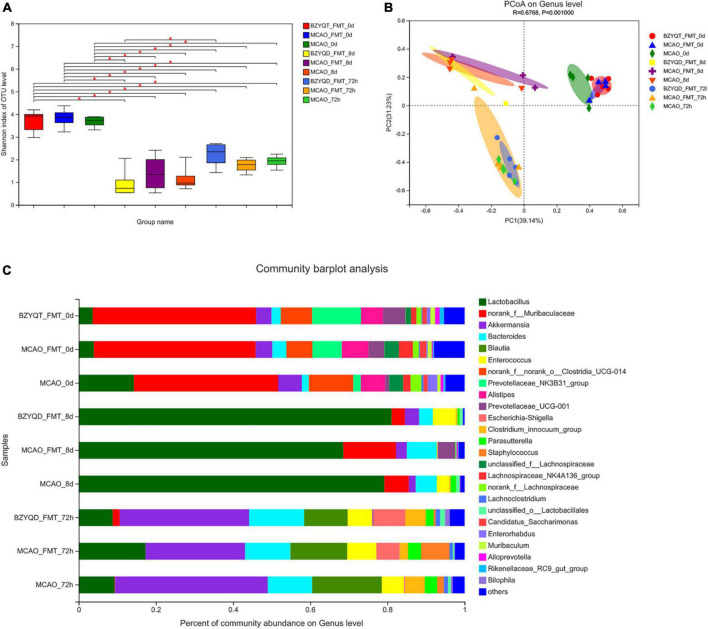
The effect of each node operation on the intestinal flora of mice in the microbiota transplantation stage. Intestinal microflora α Diversity **(A)** is the Shannon index; **(B)** is the PCoA analysis; **(C)** is the species composition at the genus level at each time point in the fecal transplantation stage. *p < 0.05. Data are expressed as mean ± standard deviation. n = 5/5/4.

After 16S rRNA analysis of the intestinal microbes of mice after antibiotic depletion, we found that compared with the initial flora, the decrease of Shannon indices indicated that the α diversity of the three groups of mice decreased sharply (Figure 5A, *P* < 0.05). The three groups were located in similar cluster positions in the PCoA analysis (Figure 5B). Cluster analysis at the phylum level showed that Firmicutes increased in high proportion in each group, and Bacteroidota decreased sharply. At the genus level, norank_f__Muribaculaceae decreased rapidly, *Lactobacillus* genus surged as the main dominant bacteria, followed by *Akkermansia, Bacteroides*, and *Enterococcus* (Figure 5C). We can obtain that after antibiotics deplete the microbiota, the species diversity and richness of the mouse gut decreased, and *Lactobacillus* was the main species.

**FIGURE 6:**
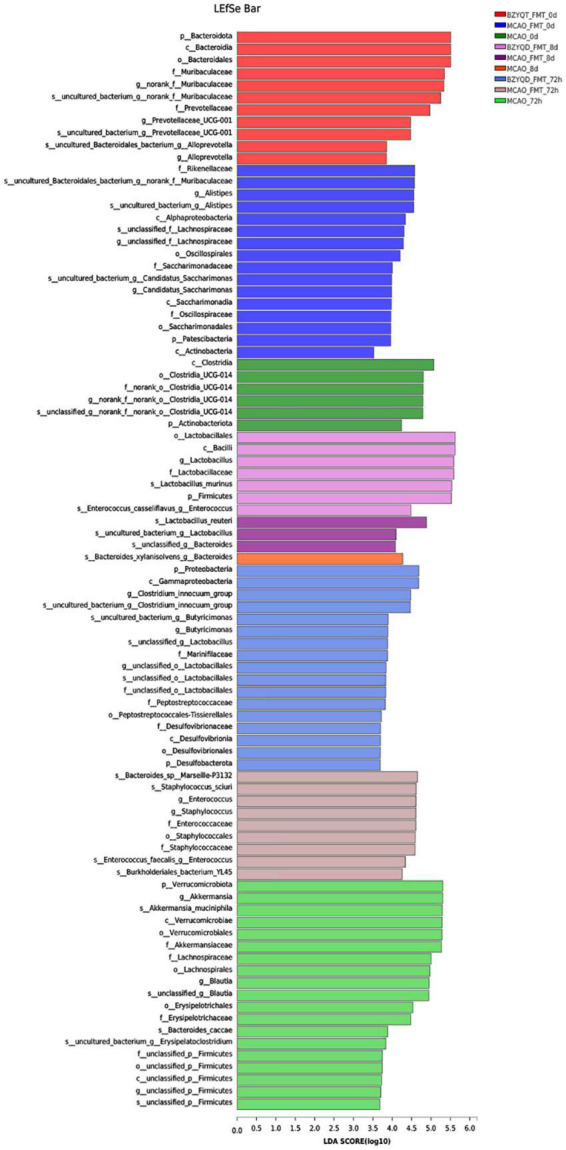
LEfSe multi-level species difference discriminant analysis for each group.

The bacterial community after 72 h of MCAO was compared with that after 14 days of prophylactic administration. The norank_f__ Roche and *Lactobacillus* were decreased in both groups. Unclassified_f__ Lachnospira, norank_f__ Lachnospira, and Brautia, *Lachnospiraceae*_UCG-006, and *Escherichia coli*–*Shigella* all increased. We used LEfSe to perform multi-level species difference discriminant analysis and found species that had a significant impact on grouping by setting the linear discriminant analysis (LDA) condition to greater than 3.5 (Figure 6). All mice were antibiotic depleted, and mice on day 0 and day 8 were almost at the same baseline. After the antibiotics depleted the flora, we could find that *Lactobacillus* and *Firmicutes* were more enriched. After the flora transplantation, we found that Firmicutes, Verrucomicrobiota, Lachnospira, *Akkermansia*, and Erysipelas were relatively enriched in the MCAO group. The harmful bacteria *Escherichia coli* and *Staphylococcus* were more enriched in the MCAO-FMT group. *Proteobacteria, Desulfovibrio, Clostridium*_innocuum_group, *Lactobacillus*, and Butyricimonas were more enriched in the BZYQT-FMT group. We found that microbiota transplantation of BZYQD-treated mice can reduce the harmful bacteria while increasing beneficial bacteria, particularly short-chain fatty acid-producing bacteria in MCAO mice.

## Discussion

The active ingredients of every single herb of BZYQD, such as mullein, ferulic acid, hesperidin, glycyrrhizic acid, saikosaponin d, have all been proven to have neuroprotective effects, and the way they exert their effects is related to anti-apoptosis. Among them, ferulic acid and hesperidin can also reduce the inflammatory infiltration of intestinal epithelial cells, protect the intestinal mucosa, and improve gastrointestinal transmission. Our data suggested that the prophylactic administration of BZYQD can improve neurological function and behavior in MCAO mice, reduce the production of apoptotic cells in the ischemic penumbra cortex and striatum, regulate intestinal flora, and increase the abundance of *Akkermansia* and *Prevotella* _NK3B31_group. The transplantation of the microflora administered with BZYQD can reproduce the above beneficial results, including improved neurological function, rotarod behavior, cerebral infarction volume, and striatal apoptotic cells, and its beneficial effect may be related to Lachnospira_UCG-006. It is related to the enrichment of *Lactobacillus* and Butyricimonas.

Prophylactic administration of BZYQD increased the abundance of *Akkermansia* and Prevotella _NK3B31_group in mice after MCAO. The abundance of these two bacteria was significantly increased compared to the control group. LEfSe analysis also indicated that these two bacteria were important different bacteria in the BZYQD group after modeling. Prevotella is considered to be a common gut commensal that mainly digests dietary carbohydrates to promote growth (Wu et al., 2011) and is an important butyrate producer (Esquivel-Elizondo et al., 2017). In addition, multiple studies have reflected the effects of Prevotaceae_NK3B31_group as a probiotic in reducing inflammation (Liu et al., 2019), regulating blood sugar, and regulating metabolism. One of the major changes in the cecal microbiome caused by experimental stroke is a marked reduction in Prevotella (Houlden et al., 2016). According to our experimental results, the neuroprotective BZYQD group Prevotellaceae_NK3B31_group level was increased, leading us to believe that this is a beneficial bacterium that can improve stroke prognosis.

*Akkermansia*, which belongs to the Verrucomicrobia phylum, is a short-chain fatty acid producer that has been extensively studied as a probiotic with outstanding anti-inflammatory properties (Zhang et al., 2017). The *Akkermansia* strain *Akkermansia muciniphila* is a symbiotic beneficial bacterium of the intestinal mucus layer, and its colonization in the cecum has been inferred to be associated with intestinal epithelial homeostasis and upregulation of leukocyte antigen presentation genes related (Wang et al., 2020). It is one of the most representative culturable strains in the phylum Verrucomicrobia, with a high content in human and animal feces (Rufeng et al., 2008). Its abundance is closely related to human health and can participate in the regulation of glycolipid metabolism and maintain the intestinal barrier (Rufeng et al., 2008; Li et al., 2021), alleviating cognitive impairment (Freitag et al., 2019). In addition, a decrement in the abundance of *A. muciniphila* in various diseases, including colitis, diabetes, etc., has been reported in several studies. In our study, prophylactic administration of BZYQD can increase the number of *Akkermansia* and occupies a higher proportion after surgery. We believe that *Akkermansia* may be an effective neuroprotective agent of BZYQD for the key producer of the action.

The low load state of recipient microorganisms is conducive to better colonization of the flora. Pre-treatment of the gut with antibiotics promoted the colonization of the intestinal mucosa by allogeneic microorganisms and increased the efficiency of FMT compared to gut-cleansing pretreated and unpretreated mice undergoing microbiota transplantation (Sarrabayrouse et al., 2020). After the antibiotics are depleted, *Lactobacillus* significantly increases, and *Lactobacillus* is extremely acid-resistant and can still survive in an environment of pH 3.0∼4.5, which not only is more conducive to the growth and reproduction of probiotics, but creates a better background for flora transplantation with a more favorable colonization environment.

In this study, the microflora used in the microbiota transplantation was the fecal granules of mice after the prophylactic administration of BZYQD for 14 days. It is also better than the control group of flora transplantation. In this part of the research on flora transplantation, it is clear that one of the key links in the preventive administration of BZYQD is the flora of BZYQD 72 h after reperfusion. The intestinal flora analysis showed that the BZYQD-FMT group contained more Lachnospira_UCG-006, and the MCAO-FMT group contained a higher proportion of *Streptococcus*, Norank_f__Erysipelas, and Lachnospira Section_NK4A136_group.

Lachnospira_NK4A136_group and Lachnospira_UCG-006 are butyric acid-producing bacteria in SCFA that are essential for intestinal barrier integrity and gut health (Hamer et al., 2008), and have been shown in multiple stroke studies beneficial effect. In clinical studies, butyric acid-producing bacteria (*Lachnospiraceae*) were reduced in the gut microbiota of patients with high-risk stroke (Zeng et al., 2019; Tan et al., 2021). The improvement of Naomaitong in ischemic stroke in mice is associated with the enrichment of the beneficial *Lachnospiraceae* (Lin et al., 2021). *Streptococcus* and norank_f__ Erysipelas are harmful bacteria. Bacteroidetes and Erysipelas were identified as biomarkers for lacunar infarction in a clinical study on gut microbiota markers in acute ischemic stroke (Xiang et al., 2020). We believe that the MCAO-FMT group promotes the growth of the probiotic *Lachnospiraceae*_NK4A136_group, which may be related to its improvement in cerebral infarct volume, whereas the growth of harmful bacteria is associated with poor stroke prognosis.

We found in the LEfSe analysis that the beneficial effects of the BZYQD-FMT group on stroke may also be related to the probiotics *Lactobacillus* and Butyricimonas. Lactic acid bacteria belong to the phylum *Firmicutes, Bacillus*, order *Lactobacillus* and *Lactobacillus* family, and produce lactic acid by fermenting carbohydrates. *Lactobacillus* has been shown to have anti-apoptotic and anti-inflammatory effects on it. Administration of three mixed probiotics including *Lactobacillus* can inhibit neuronal apoptosis through benign regulation of apoptotic factors in the hippocampus (Xu et al., 2018). More than a dozen *Lactobacillus* strains can modulate anti-inflammatory responses by increasing the production of TNF-α, IL-1β, IL-10, and IL-6 by immune cells (Zieliñska et al., 2019). According to a study, the improvement of neurological function in stroke rats by bone marrow mesenchymal stem cells (BMSCs) is related to the increased abundance of *Lactobacillus* (Zhao et al., 2021). Naomaitong relies on gut microbiota to improve stroke outcomes in rats with a significant increase in *Lactobacillus* abundance during stroke prognosis (Lin et al., 2021). The anti-inflammatory effect of atorvastatin was associated with increased intestinal *Lactobacillus* levels in a mouse study of stroke treatment (Zhang et al., 2021). Therefore, *Lactobacillus* is a beneficial element in ischemic stroke.

Butyricimonas is a kind of butyric acid-producing bacteria. Butyricimonas and other butyric acid-producing bacteria ferment with dietary fiber in the intestinal lumen to produce butyrate (Barcenilla et al., 2000). Butyrate is the primary energy source of choice for colonic epithelial cells in all SCFAs (Barcenilla et al., 2000) which plays an important role in the regulation of immune and inflammatory responses (Flemming, 2019), intestinal barrier, and intestinal homeostasis (Tan et al., 2014). Butyrate is a major member of SCFA, and bacteria that produce SCFA and butyrate can regulate the peripheral and central nervous system and brain function (Bourassa et al., 2016). Treatment of stroke mice with short-chain fatty acid-producing bacteria can significantly promote post-stroke recovery, regulate immunity, and affect microglia (Lee et al., 2020; Sadler et al., 2020). Transplantation of fecal bacteria rich in short-chain fatty acids and butyric acid supplementation can both treat ischemic stroke by improving intestinal microbiota (Chen et al., 2019). Butyricimonas were less frequently studied in ischemic stroke but were enriched in a study of FMT to promote neurological recovery in mice with spinal cord injury (Jing et al., 2021), and inversely correlated with pro-inflammatory gene expression (De Filippo et al., 2010). Probiotics were shown to be associated with improved post-emergency cognitive impairment in a clinical study that significantly promoted the enrichment of Butyricimonas spp (Bloemendaal et al., 2021).

With the in-depth study of intestinal flora, the importance of intestinal flora has been more and more confirmed. It has been reviewed that stroke is associated with a change in gut flora, which indicated that intestinal microflora may be a new target for the prevention and treatment of ischemic stroke. Meanwhile, Chinese herbal medicine was demonstrated to improve various diseases, such as coronary heart disease, depression, tumors, etc., by regulating intestinal flora. However, there are few studies about the effect of traditional Chinese herbal medicines on intestinal flora in rats with stroke. In this study, we explored the regulatory effect of BZYQD on intestinal flora and the protective effect of BZYQD on brain nerves, which provides new ideas for the treatment of stroke and is worthy of in-depth study.

Studies have reported that the intestinal microecology of mice is affected by age, intestinal motility, pH, diet, and environment and varies according to its location (Turnbaugh et al., 2009; Gu et al., 2013; Lee et al., 2020). The cecum, colon, and feces are farther from the stomach and are less affected by transient environmental changes. Although fecal microbial diversity is lower than the former two, the three share more core microbiota, and their diversity is more similar (Gu et al., 2013; Lkhagva et al., 2021). Therefore, in the experiment, we used the cecum and fecal pellets to detect intestinal flora, which may have some limitations. In addition, in the microbiota transplantation stage, a control group was not set up to analyze the effect of antibiotic depletion on the experiment, and the intestinal microbiota of the donor mice in the microbiota transplantation stage was not detected, but the intestinal bacteria that had been prophylactically treated for 14 days in the previous stage were selected. The comparison of results is another limitation of this experiment. In addition, we should recognize that the MCAO model is useful for studying the dynamics of microbiota changes after stroke, but is relatively homogeneous for ischemic stroke in humans. Ischemic stroke can be combined with a variety of unhealthy factors in the human body, such as hypertension, diabetes, hyperlipidemia, and atherosclerosis (Virani et al., 2021), and these states are bound to have a certain impact on gut microbes. We encourage other researchers to continue to study the impact of gut microbes on ischemic stroke. These studies will establish a more complete gut microbiota database for ischemic stroke and provide insights into disease-related driver bacteria and targets for disease treatment, even laying the foundation for bacterial research.

## Ethics statement

This animal study was reviewed and approved by Animal Research Committee, Laboratory Animal Center, Shanghai University of Traditional Chinese Medicine (PZSHUTCM18110603).

## Author contributions

QL and MC wrote the first draft and incorporated contributions from all co-authors into the final article, which was approved by all authors prior to submission. QL, MC, and ML conducted animal studies and collected data. QL and MLL carried out the high-performance liquid phase detection of traditional Chinese medicine compounds. QL, MC, YCZ, JM, and ZJW analyzed the data results. YYZ designed the entire study and received funding. YYZ and ZFW guided and planned the whole experiment. All authors contributed to this article and approved the version submitted.

## Footnotes

1
https://mothur.org/wiki/simpson/

2
http://www.microbesonline.org/fasttree/
