# Supplementary figures and images for: The protective effect of Buzhong Yiqi decoction on ischemic stroke mice and the mechanism of gut microbiota
Source: Front Neurosci. 2022 Dec 15;16:956620. doi: 10.3389/fnins.2022.956620 (PMC9798918; doi:10.3389/fnins.2022.956620)

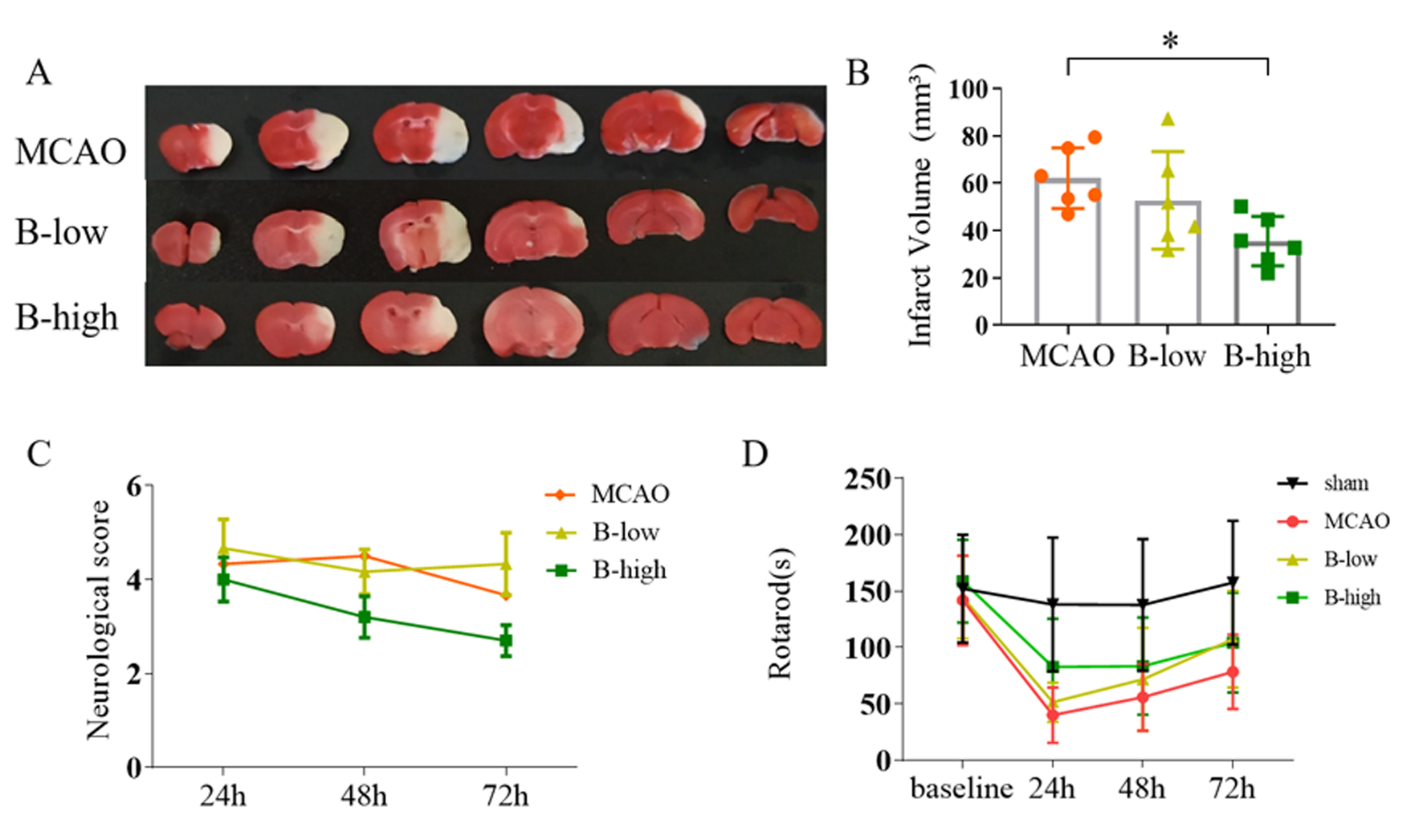

Supplement: Supplementary file 1 [file Image_1.TIF]
